# The complexity of rural contexts experienced by community disability workers in three southern African countries

**DOI:** 10.4102/ajod.v4i1.167

**Published:** 2015-06-09

**Authors:** Margaret Booyens, Ermien van Pletzen, Theresa Lorenzo

**Affiliations:** 1 Department of Social Development, University of Cape Town, South Africa; 2 Centre for Higher Education Development, University of Cape Town, South Africa; 3 Department of Health and Rehabilitation Sciences, University of Cape Town, South Africa

## Abstract

An understanding of rural communities is fundamental to effective community-based rehabilitation work with persons with disabilities. By removing barriers to community participation, persons with disabilities are enabled to satisfy their fundamental human needs. However, insufficient attention has been paid to the challenges that rural community disability workers (CDWs) face in trying to realise these objectives. This qualitative interpretive study, involving in-depth interviews with 16 community disability workers in Botswana, Malawi and South Africa, revealed the complex ways in which poverty, inappropriately used power and negative attitudes of service providers and communities combine to create formidable barriers to the inclusion of persons with disabilities in families and rural communities. The paper highlights the importance of understanding and working with the concept of ‘disability’ from a social justice and development perspective. It stresses that by targeting attitudes, actions and relationships, community disability workers can bring about social change in the lives of persons with disabilities and the communities in which they live.

## Introduction

With their rights, talents and potential to learn too often overlooked, persons with disabilities form a structurally disadvantaged group in society, who are excluded from opportunities to participate actively in community life. When people are excluded from the social and economic life of their community and country they find it difficult to meet their own needs. This exclusion, in turn, places pressure on the government, non-governmental organisations (NGOs) and the families of persons with disabilities to provide support and services. Internationally, the focus has shifted from institutional or clinic-oriented rehabilitation services to the deployment of community disability workers (CDWs), whose main aim is to facilitate the social inclusion of persons with disabilities (WHO 2010) to ensure that their needs are addressed. The purpose of this shift is to empower persons with disabilities to advocate for themselves, as far as they are able, their right to participate in all facets of community life.

A number of factors, at individual, family, community, local, national and global levels, feed into the processes leading to poverty and the social exclusion of various social groups. These factors can be used to create an integrated framework for analysing social exclusion and poverty (Burchard, Le Grand & Piachaud 2002, cited in Haralambos & Holborn 2008). We have used this framework to analyse the experiences of a sample of CDWs. We pay particular attention to the community context which, though rural, is nevertheless affected and shaped by factors at all levels, right up to global level.

Our motive for selecting rural communities is that persons with disabilities are particularly vulnerable and disadvantaged in rural contexts and, also, in some high density areas in urban settings. Typical discriminatory attitudes to and beliefs about disability are exacerbated by income poverty, power dynamics, a dearth of needed resources and the consequent competition for the few available resources (Rule, Lorenzo & Wolmarans 2006; Van Pletzen, Booyens & Lorenzo 2014). The result is social exclusion for the disabled. Beyond the issue of income, poverty can also be more broadly understood as a lack of the power and resources to make choices and take advantage of opportunities (Davids, Theron & Maphunye 2009). Poverty is deeper and more widespread in rural than urban areas. In conducting this research we could clearly see the damaging effect of different forms of poverty on all aspects of the lives of individuals, families, households and whole communities (see also Van Pletzen *et al*. 2014).

There are more persons with disabilities in rural than in urban areas (ILO 2007) as, in these areas, they are less likely to be socially mobile, yet research on disability in rural areas remains sparse, particularly concerning the challenges of rural community disability work. Duncan, Sherry & Watson (2011:30) observe that rurality ‘is more than a geographical concept’. They expand the meaning of the term to include ‘the structure, state and quality of life of people living in sparsely settled places away from the direct influence of large cities and towns’. Rural dwellers tend to be isolated from information, the media, networks, contact with other people and resources such as education and health services (Lorenzo & Cloete 2004; Lorenzo, Van Pletzen & Booyens 2015). But despite this isolation they are nevertheless directly influenced by government policy and legislation, corruption in the public and private sectors, and global factors such as an economic recession.

The Convention on the Rights of Persons with Disabilities states the following about disability:

[*D*]isability is an evolving concept and results from the interaction between persons with impairments and attitudinal and environmental barriers that hinder their full and effective participation in society on an equal basis with others. (UN 2006:1)

This understanding of disability resonates with Burchard *et al*., whose integrated framework (2002, cited in Haralambos & Holborn 2008) indicates that it is people's interaction with the various systems in their environment – a rural context in our case – that generates the experience of disability, with social exclusion being a fundamental and well-documented feature internationally.

Working within a human rights perspective advocated by the World Health Organisation's community-based rehabilitation guidelines (WHO 2010), CDWs are encouraged to identify, create and use opportunities to promote the inclusion and participation of persons with disabilities in the social and economic life of their communities and country. By removing barriers to community participation, they will enable persons with disabilities to satisfy their fundamental human needs. However, insufficient attention has been paid to the challenges that rural CDWs face in trying to realise these objectives. Most studies to date have focused either on the role of the CDW or on the needs of the disabled person. This study, therefore, sought a deeper understanding of the complex realities presented by rural communities and the skills needed by CDWs working in these contexts.

## The country contexts in relation to human development

The research was conducted in Botswana, Malawi and South Africa in 2011 and 2012. Botswana is one of the world's most sparsely populated countries, with just under two million people, 41.2%, living in rural areas. It has a Human Development Index (HDI) of 0.633, an improvement on the 0.446 recorded in 1980 (UN 2011). Malawi has a population of 13.9 million, around 82% of whom live in rural areas (UN 2011). Its HDI improved from 0.270 in 1980 to 0.400 in 2011, but the country remains in the ‘low’ human development category. South Africa, like Botswana, has an HDI that places it in the ‘medium’ bracket, with an improvement from 0.564 in 1980 to 0.619 in 2011. Its population is just over 50 million, with 39.7% living in rural areas. Life expectancy at birth in Botswana, Malawi and South Africa is 53.2, 54.2 and 52.8 years respectively (UN 2011).

Over a period of nearly 15 years, SINTEF studies on living conditions of persons with disabilities have been carried out in Botswana, Malawi, Lesotho, Zimbabwe and Zambia (see http://www.safod.org). A regional study of the Eastern and Western Cape in South Africa was conducted in 2006 (Loeb et al. 2008). A review of all these studies has demonstrated that the pattern of difference is consistent: individuals and households with a member with a disability experienced substantial gaps in access to services, social and economic status, access to information and to assistive devices and social participation (Eide & Ingstad 2013). Persons with a disability experience lower levels of living, particularly those in rural areas, and women with disabilities are worse off than their male counterparts. This translates into a denial of equal opportunities for persons with disabilities to participate and contribute in their community and society, which is a violation of their human rights.

## Research methodology

Botswana, Malawi and South Africa were selected to add to the knowledge base of policy makers and service providers on contextual issues that influence the inclusion of persons with disabilities in communities in southern Africa. In addition, they were the home countries of the four postgraduate students taking a Diploma in Disability Studies at the University of Cape Town, who undertook the fieldwork and data gathering as a requirement of one of their courses. This qualitative study was based on a purposive sample of 16 experienced CDWs working in rural communities in these three southern African countries.

The selection criteria for the purposive sample of CDWs were as follows. Participants needed at least five years’ experience of community disability work in rural areas, and there had to be a balance of male and female participants from a range of ages. We also selected a mix of people who had a disability, either personally or in their family, and others who did not.

The profile of the participants (using pseudonyms) is set out in Table 1. The 16 CDWs who participated in the study were eight women and eight men, ranging in age from 26 to 54 years. Four of them had an impairment, one as a child, whilst 10 had a family member with a disability. Their years of experience in working in the field of disability ranged from five to 27 years. The majority were in paid positions in Ministries or Departments of Health in various job categories, including Social Work and Community Development. Four worked for NGOs.

**TABLE 1:** Profile of participants (community disability workers).

Pseudo-nym	Sex	Age	Employer	Job title	Experience (in years)	Living with own disability	Disability in family
Botswana							
Odirile	F	54	MoH	Family welfare educator	22	No	Sister with temporary disability after poisoning incident; child with spinal injury
Lily	F	35	MoH	Family welfare educator	10	No	Nephew (not specified)
Mothusi	M	34	MoH	Family welfare educator	11	No	No
Karabo	F	54	MoH	Family welfare educator	27	No	Granddaughter (not specified)
Masego	F	46	MoH	Rehabilitation officer	13	No	Daughter blind, in wheelchair, intellectually disabled
Gil	M	36	NGO	Rehabilitation technician	12	No	Father had stroke
Kgomotso	F	45	MoH	Rehabilitation officer	17	No	Child with convulsions for first 3 years of life; blind cousin (fully integrated)
Kefilwe	F	31	MoH	Social worker	7	Yes	No
Malawi							
Jimu	M	46	MoH	Rehabilitation officer	17	Yes	Cousin, intellectually disabled, blind
Emak	M	36	NGO	Rehabilitation technician	10	No	Father, psychiatric illness; relative disabled by polio
Nick	M	40	NGO	Rehabilitation technician	17	No	No
Chisomo	M	26	NGO	Rehabilitation technician	5	No	No
South Africa							
Noname	F	35	DoH	Community development worker	5	Yes	Brother, intellectually disabled
Jeffrey	M	45	DoH	Occupational Therapy technician	17	No	No
Londi	F	43	DoH	Occupational Therapy technician	20	Yes, as child	Brother (not specified, but needs care as adult)
Mpho	M	45	DoH	Occupational Therapy technician	10	No	No

The CDWs who participated in the research had a variety of job titles (see Table 1). They are all referred to as ‘community disability workers’ in this article. This broad term encompasses a range of levels of education and training and of roles that extend beyond rehabilitation to embrace prevention, education, community development and policy practice^1^.

### Data collection

One in-depth interview was conducted with each CDW, which was divided into two parts of about an hour each, digitally recorded and transcribed. The interviewers each recruited four CDWs and conducted interviews with them in English, at places and times convenient to the CDWs in their home country.

The interviews elicited the following details about the CDW:

- their childhood experiences
- specific events that influenced their choice of career
- low and high points in their work with persons with disabilities
- the biggest challenge they faced in working with disability issues
- the person or organisation that had taught them most about disability and development.

This article draws on the CDWs’ descriptions of and reflections on the contexts in which they performed their community-based rehabilitation work.

## Data analysis

Thematic content analysis was undertaken by a research team of academics from a range of backgrounds, and also by two of the students who conducted the interviews. The team read the transcripts independently and then met for the analysis process. Two groups of four people analysed one transcript per group and then discussed all the emerging themes. Fifteen themes were agreed upon. Following this four pairs each analysed one additional transcript. Five more themes emerged. The 10 remaining transcripts were then assigned to individual members of the team to analyse, using the expanded framework of themes. No new themes emerged, which suggested that a data saturation point had been reached. The group then identified two superordinate themes: the personal and the context. The latter theme is the topic of the current paper, the personal themes having been dealt with by Rule, Kahonde & Lorenzo (2015). For context, core themes were further identified by grouping the sub-themes that had been initially identified through consensus discussions. Dedoose, a software programme for qualitative data analysis, was then used to analyse the relationship between the identified themes. Post-analysis, the students who had conducted the interviews undertook member checking. They went back to the participants, either telephonically or in person, to check whether or not the thematic framework identified by the research team was consistent with the information they had provided during the interviews.

Whilst the study opens a window on the day-to-day realities of a small group of CDWs working in rural communities in three southern African countries and provides useful insight within the region, the findings are specific to these contexts. A further limitation is that the postgraduate students lacked extensive research experience at the time of data gathering, which may have affected the quality of their first interviews. With feedback from the academics between the first and second parts of the interviews, their interviewing skills improved substantially.

## Results

Thematic content analysis led to the emergence of five main themes related to the rural context of the CDWs work:

- discriminatory attitudes
- the impact of poverty
- difficulty accessing community resources
- local governance
- the influence of donors.

These themes are presented with supporting quotations from the interviews.

### Discriminatory attitudes

Families, the building blocks of society, constitute the basic system or context in which individuals attempt to meet their needs. Family dynamics can, however, work to exclude members with a disability, causing stress to all family members. Our participants identified discriminatory attitudes, beliefs and actions of family members as significant problems for persons with disabilities. These included rejection, acceptance of the person but not helping them develop their full potential, and feelings of shame and consequent hiding of the person with disabilities:

‘It pains me a lot when I see a parent is in the forefront trying … to give names to his or her own child. Like *galu* [dog]. Is he a dog? ‘You cannot do anything, you are a disabled’ … So, I find it sometimes a big challenge to change people's attitudes’. (Nick, Malawi)

‘I think sometimes they get satisfied with the way the child is. They just have the feeling that there is nothing that can be done with that child. It's a kind of belief’. (Chisomo, Malawi)

‘The other situation is when I see most people hiding persons with disabilities, not taking them out. It really pushed me to make a difference in the life of persons with disabilities’. (Mpho, South Africa)

There was evidence that in some instances families not only hid disabled family members but actually left them to their fate:

‘The most prominent low point in my life … was when I realised that some families had given up hope on their disabled family members and abandoned them as useless and futureless street beggars’. (Mothusi, Botswana)

The findings also indicate that some family actions foster an exploitative and opportunistic type of inclusion, for example using a young disabled person to generate an income for the family by begging along the roadside.

In response to such attitudes, the participants spoke about how they had facilitated the inclusion of persons with disabilities within families using demonstration, education and counselling strategies to foster changes in attitude and practice within the family context itself. However, CDWs often find the process an uphill struggle, and frustrating when their efforts are thwarted, sometimes by parents. Emak, a Malawian CDW, recounted how some parents will ‘wear a face with a positive mind’ during contact at his office but will subsequently fail to ensure that their disabled child uses the allocated equipment at home.

Work with individual persons with disabilities can lead to positive changes in their self-image and in their ability to engage in collective action in an attempt to change their circumstances. Kefilwe, a Botswanan CDW, explained:

‘I have some people whom I have groomed and now they have grown and now they are voicing out [*speaking out*]. They are even part of the youth who are advocating for the rights of persons with disabilities’.

Discriminatory attitudes towards persons with disabilities within families and communities are self-reinforcing. The strength of these attitudes in rural areas may link to culture-related superstitious beliefs about persons with disabilities, and indeed the beliefs of persons with disabilities about themselves:

‘The biggest challenge is when persons with disabilities are stigmatised, marginalised and not respected, are not given opportunities to make decisions. They are not taken seriously like other people. It is still a challenge because the community still thinks that they cannot do anything for themselves’. (Mpho, South Africa)

‘At that time, you know, there were these primitive beliefs that if you get in touch with them [*persons with disabilities*] you will get children with disabilities … A lot were suffering out there in the bush without help, refusing to go to the hospitals, believing that they can sort their disabilities by curing them traditionally to get rid of witchcraft or whatever they believed in’. (Kgomotso, Botswana)

The experiences of the CDWs suggest that living in a rural community poses serious challenges to persons with disabilities. It limits their integration into community life and restricts their access to the resources they need, in large part resulting from discriminatory attitudes, traditional belief systems and a shortage of resources. In working to promote the social inclusion of persons with disabilities, our participants had to spend much time and energy combatting negative attitudes and building relationships, with the expectation of ultimately bringing about social change.

#### The impact of poverty

The participants encountered various forms of poverty, which, together with rurality, compounded the difficulties experienced with participation and inclusion by persons with disabilities.

Income poverty makes it difficult for parents to provide the level of care they intend for their disabled child. This situation is especially stressful for the parents of children with disabilities who have particular needs, for example, nutrition:

‘You will find out that particularly those with cerebral palsy … some are not able to eat anything that is eaten in the family. They need some special diet. And you can imagine how you would feel – that there's this poor soul … they have the right to live but the parents do not have the means to give proper care – they wish they could but they don’t have the means’. (Masego, Botswana)

In their efforts to generate an income, some parents cannot find the time and money to enable their disabled child to benefit from a care facility: ‘They can’t find time to bring children to our centre. They keep looking for piece (*any*) work from different people and also doing business’. (Chisomo, Malawi)

A South African participant spoke of families not accessing existing resources such as the government disability grant because they simply did not know about it. In one instance, the participant had written information on the disability grant, but because she was illiterate, she did not know what steps to take to access it: ‘He was staying with a very old … grandmother [*who*] did not have knowledge about the grant and that family was poor’. (Noname, South Africa)

An important part of our participants’ work, as community disability workers, was, thus, to communicate information or to link families to networks through which they could access information. In Malawi, for example, parents’ associations serve this networking function.

It is critically important that, especially when working in contexts of poverty, CDWs move beyond rehabilitating individual persons with disabilities to actively promoting their participation in the economy of the community and the country. Two of our participants were clearly encouraging this:

‘Some of them, after taking them for some course like in [*name of centre and town*], when they came back some … started their own gardens – some were tailors and some were poultry keepers’. (Masego, Botswana)

‘I wanted a person with a psychiatric disorder to work with a physically disabled person starting a car wash because the grant is little for them [*persons with disabilities*] … I also motivated for the local lodges to bring their cars … even in the schools I asked all teachers to bring their cars. So they started to realise that they can work for themselves. Even the community started to respect and love them. They are not going around begging for money from people but they work for themselves’. (Londi, South Africa)

#### Difficulty accessing community resources

Our participants spoke about how difficult it was for persons with disabilities, their families – and indeed themselves – to access, good quality or even minimally adequate educational, transport and health resources in the rural areas in which they lived and worked.

**Education systems that exclude**: The poor quality of education offered at many rural schools affects the non-disabled youth and even more so the disabled. A participant described the quality of the teaching available in local schools:

Those teachers were worse, worse, worse, even in secondary we still had some unqualified teachers … [*At primary* [*school*], it was just the JC [*junior certificate*] school leavers, some of them old standard six teachers. [*At*] secondary school we still had some form five dropouts [*teaching us*] who had never been to the university. (Kgomotso, Botswana)

Income poverty constrains both access to education and the completion of schooling. Several participants mentioned young people who had dropped out of school because of poverty. In rural areas, access to sparsely distributed schools requires transport, with related cost implications. In the case of children and young people with disabilities who require special schools, for whom integrated schools are not suitable, commuting is not an option. They must become boarders in one of the few towns or cities where these schools are located.

**Inaccessible public transport systems:** The public transport systems in rural areas were identified as a significant barrier to persons with disabilities, preventing them from meeting both their health related and educational needs. Although not specifically mentioned by the participants, an inadequate transport system would also hinder access to social visiting, sporting and other community activities. The distance to resources, the cost of transport, the lack of vehicles with access for persons with disabilities, and the insensitivity of some public transport drivers, put obstacles in the way of school completion, and made it difficult for persons with disabilities to access hospitals for treatment and rehabilitation: ‘[*Trips to hospital are*] also expensive for them because they are supposed to appoint personal assistants for coming here, so they pay more than expected’. (Jeffrey, South Africa)

Transport problems also hampered the participants in their work:

‘I wish all people with disability can be assisted but the shortage of transport and manpower is still a problem. I feel like my work is incomplete. I really need these problems to be solved’. (Odirile, Botswana)

One participant exhibited the power of creative thinking in her strategy for sensitising taxi drivers to the needs of persons with disabilities:

‘We put them [*the taxi drivers*] in wheelchairs being blindfolded, and we put marshmallows in their mouth for them not to talk. So we used [*drove*] their taxis and they were passengers. We made sure that if they want to step out in the street, for example the hospital gate, we just pass for a little distance because we wanted them to feel the pain, as blind people do when you drop them’. (Londi, South Africa)

**Poorly funded health systems:** The funding of health services was a recurrent theme in the 16 interviews, particularly with regard to the way it obstructed access to good health services for the disabled. In the context of the global economic recession and a situation of competing demands for health spending by governments and international aid agencies, NGOs and indeed government health services are finding it very hard to meet the health needs of the people whom they serve:

‘You really think no, we will consider this when funds are available and it's like they will never be available because now we have been hit by recession and another one is said to be coming, so I don’t think it will ever be given priority’. (Masego, Botswana)

Gil, a Botswanan participant, said that a particular NGO had insufficient funds for equipment:

‘The challenges are finances … The organisations that we are working with … you find that they don’t have the muscle power … You find that the medical equipment is too expensive’.

The absence of rehabilitation facilities for children was mentioned by two participants: for autistic children in rural areas of Malawi, and for blind children and children with multiple disabilities in Botswana. Another commented on the local hospital's failure to budget for assistive devices:

‘Another challenge is that of assistive devices. When you request to the hospital manager, they say ‘no budget’. It is difficult sometimes to work as a rehabilitation worker’. (Emak, Malawi)

Other participants highlighted the need for parents of children with disabilities to have a respite from their constant, demanding roles as carers – but pointed to the absence of respite care facilities. This finding raises the question of how much practical support health systems provide in rural communities for the special needs of family members and other household members caring for the disabled.

The participants were keenly aware of and frustrated by the insufficiency and the inadequacy of the resources available to persons with disabilities and their families living in rural communities. There was evidence of how they used their community development competencies to address some of the access related difficulties, such as the insensitivity of some taxi drivers. It was also evident that some participants were facilitating the development of networks between service providers as an intervention to help address the needs of persons with disabilities. This is illustrated in the section that follows.

## Local governance

In the rural communities that were the subject of our study there were intricate forms of political leadership, including both traditional authorities and elected politicians. CDWs need to understand and be capable of engagement with these different types of authority and the power they exercise. The desire of politicians to garner votes can lead them to take misguided actions. For example providing aids to persons with disabilities without first assessing their needs. However, given the power that they wield, our participants sometimes found it difficult to confront them:

‘The politicians take those vulnerable people as ground for campaign … they go there and find that there are people with disabilities. What they think is to bring wheelchairs and tricycles and supply to them without proper assessment. So if you go there and say that this is not necessary for you, they [*persons with disabilities*] would say that it is the MP [*Member of Parliament*] who gave us this thing. So an MP is someone with high ranking in the community. You are also under the same MP. To go there and confront him is not easy at all’. (Emak, Malawi)

A particular challenge our participants faced was deciding what action to take when leaders abused their positions of power and violated the rights of persons with disabilities for personal financial gain. One participant (country withheld to protect identity) recounted how the executive director of an agency had sold items meant for children, using the proceeds for her political campaign. She had become a Member of Parliament.

A Malawian participant offered an example of the inappropriate use of power by traditional leaders:

‘When we went to the village to train them [*traditional leaders*], they put money in here [*they brought money into the picture*]: ‘So unless you give us [*money*], we are going to be very passive’. This is an issue concerning their people, their subjects, but then they put [*bring in the issue of*] money … so I ended up reporting him to the traditional authority’. (Nick, Malawi)

Adding another dimension to community complexity is the range of ethnic groupings and belief systems, which may present barriers to the inclusion of persons with disabilities:

‘This work is very difficult to change someone, family or the community because of different beliefs and here in [*name of town*] there are many tribes, churches, traditional beliefs and they do differ so it needs more effort’. (Odirile, Botswana)

The influence of traditional leaders on attitudes towards persons with disabilities adds to the complexity of the dynamics in rural communities:

‘Yes, stigma was there. I remember one time, somebody refused to take water from the cup which that girl used. So that was a kind of discrimination. And I remember we were trying to find out why. But the elders [*traditional leaders*] said ‘’just leave it’’’. (Emak, Malawi)

The importance and power of CDWs working in positive partnerships with traditional power holders to promote opportunities for the social inclusion of persons with disabilities came across clearly in these comments from a Malawian participant:

‘I go to the villages and identify those children with orthopaedic conditions which can be corrected surgically … I need to mobilise the traditional authorities. I befriend them and tell them my objective of going to their areas. And all the things which they need to know … they call the group village heads together and I address them in front of their traditional authorities. And from there, the group village heads talk to their village heads about this project. And then later on, I send them the dates so that they should announce in several villages … someone is coming here to look at our kids who are disabled, that have orthopaedic conditions. So I have worked with them and I am still working with them’. (Nick, Malawi)

The rural CDW's role extends beyond mobilising politicians and traditional leaders to developing processes to include persons with disabilities. The CDW must also work to influence the attitudes of power holders to persons with disabilities. Whilst politicians anywhere in the world may undertake ill-conceived actions to garner votes, our findings confirm that it is particularly in rural areas that traditional beliefs influence attitudes, relationships and actions, serving to increase the difficulties persons with disabilities face in trying to meet their needs.

## The influence of donors

Our participants noted that donor funding affected their work because it had a direct effect on the quality and quantity of resources available to communities, especially in the current global economic recession. The problem was not just a matter of the adequacy of resources; the donors’ expectations and priorities could also create problems for CDWs.

The study revealed that the donor of a Malawian NGO preferred the NGO to supply evidence of working with large rather than small numbers of persons with disabilities. The donor evidently did not share the participants’ insight and understanding that lower statistics possibly pointed to the effectiveness of services:

‘You find that when you come up with less statistics, they question you. I remember … we had a meeting in [*name of town*] … and there was an auditor sent by the donors. They asked me ‘Why are your statistics low?’ I said I have to be proud of that. They asked ‘Why?’ I said, if I had a lot of persons with disabilities … it means that primary health care is not done … But they were not happy with that. They wanted big statistics … I was surprised. Why do you want [*big*] statistics? You want the people to suffer, so that you can give us money. No, that is not [*good*]’. (Emak, Malawi)

Another Malawian participant recounted how a northern association for persons with disabilities had switched from providing assistive devices to only funding repairs of the devices. His perplexity is reflected in his question: ‘But my question is, why can you say repair? Repair what, if you do not provide the assistive devices?’ (Jimu, Malawi).

Jimu recounted how another donor requested the recipient Malawian NGO to stop paying for the secondary schooling and college fees, of young persons with disabilities, resulting in some having to drop out. He also told how a donor had refused to fund one type of disability, selecting another, saying that the changes brought about through services to the latter were more visible:

‘But when I was working at [name of organisation], they refused to fund work with cerebral palsy because they said that they can’t see a change and opted for those with blindness because they could see the change. But with cerebral palsy, I see a lot of changes’. (Jimu, Malawi)

The participants painted a picture of the power that donors wield in their relationships with recipient organisations. The frustration they experienced in their attempts to influence these relationships in the context of cash-strapped, under-resourced rural communities was evident.

## Discussion

Overall, the CDWs in this study demonstrated steadfast commitment to promoting the social inclusion of persons with a disability. What makes this remarkable is that they were clearly not being given the support they needed whilst working in communities that present daunting problems and challenges.

Our findings point to the complexity of the rural context in which CDWs work, and persons with disabilities live. A range of opportunities for and barriers to the social inclusion of persons with disabilities in family and community life emerges in our study. The particular complexity of rural contexts becomes evident when one uses the integrated framework of Burchard *et al*. (2002, cited in Haralambos & Holborn 2008) to analyse the interaction both within and between the various systems. There are numerous interrelated challenges that make up a complex context in which to work towards the inclusion of persons with disabilities and ultimately facilitate social change. Much research still needs to be undertaken to understand some of these interrelationships better, for example the potentially compounding effect of poverty on discriminatory attitudes. The gap is indeed wide between the goals for the social inclusion of persons with disabilities, set by the Convention on the Rights of Persons with Disabilities (UN 2006) and the CBR Guidelines (WHO 2010) on the one hand, and the contextual realities of community disability practice in rural areas of developing countries on the other.

Family attitudes and behaviour prevent the persons with disabilities from developing to their full potential and doing what is in their capability to do in order to meet their own needs. Together, discriminatory attitudes of family members and others towards persons with disabilities, various types of poverty and inappropriately used power, in relationships between people and organisations at various levels, present formidable and interlinked barriers to participation. Similar barriers are described in the typology of obstacles, of Barberton, Blake & Kotze, to poor people seeking to challenge power imbalances (1998): obstacles of location, resources, organisation and attitude. CDWs and generic community development workers have much in common, both groups targeting these various obstacles to participation.

Describing the limitations of development strategies, Krznaric (2007) notes that:

[*Development strategies*] display an overwhelming focus on individual actors, organised social groups, and institutions, with little acknowledgement that societies and institutions are composed of human relationships that are a potential locus of change. (p. 44)

However, disability brings into development work a specific aspect of the attitude obstacle. Discriminatory attitudes towards persons with disabilities, leading to exclusion from families and communities, are fuelled by cultural beliefs about persons with disabilities that are particularly strong in rural communities. Our study leads us to question whether or not generic community development workers are sensitive to this reality and are equipped to deal with it.

These discriminatory beliefs determine how and to whom organisations allocate resources, which are increasingly limited, resulting from the economic situation globally and in the countries concerned. Organisations, both public sector and not-for-profit, lack sufficient resources to meet the needs of those whom they serve. The importance of forming or joining networks with other service providers, to develop collective power through collaboration and partnerships, is evident and community disability workers make significant contributions in this regard (Lorenzo et al. 2015).

Issues of poverty dominate all forms of development work in rural communities. Disability exacerbates the problems and, ironically, poverty alleviation measures often exclude disabled individuals – by default, if not by intent. So many of our participants were making an effort to advocate generally for their clients’ inclusion in mainstream community development initiatives, although they could not always take proactive action to address the particular circumstances of each one. Poverty, defined from any perspective, points to fundamental human needs not being met (Max-Neef 1991). Micro-level interventions can help people to meet their basic sustenance needs but are insufficient to foster the social inclusion of persons with disabilities. CDWs need to educate persons with disabilities, their households, families and communities about their rights, and help them to access those rights. They need to mobilise and capacitate them to exercise their power collectively to influence policy and participate in decision-making for the allocation of resources at organisational, community and broader levels. Drawing on Korten's classic text (1990), we can postulate that the active participation of persons with disabilities, in the organisations that serve them, could encourage those organisations that focus on a relief and welfare type of service delivery to become more involved in community development, in influencing policy and in joining relevant global social movements. This points to the need for CDWs to facilitate the building of alliances and partnerships.

It is essential that CDWs view donor organisations and their contribution to development in a very critical light, as some of the participants, in this research, indeed did. The findings offer evidence of the power of donors and the (sometimes negative) impact of their decisions on local organisations and the persons with disabilities whom they serve.

Organisations for persons with disabilities need to give careful thought to the extent to which they should rely on donor funding. A community based rehabilitation approach necessitates holistic local resource mobilisation, rather than simply relying on ‘fundraising’ (WHO 2010).

Opportunities for persons with disabilities to participate in the economy, in other words, to work, are vital to alleviate income and other forms of poverty. Interventions that successfully include persons with disabilities in the economy of the community can help to reduce family stress, increase the self-esteem and independence of persons with disabilities, and promote positive community attitudes.

Poverty and the dynamics between power holders were found to be cross-cutting issues that add to the complexity of the rural context, compounding the difficulties in meeting the needs of persons with disabilities, and confounding the efforts of CDWs to promote their social inclusion in communities. Power is at the heart of all relationships. At times, promoting the social inclusion of persons with disabilities requires tapping into the persuasive power of important community stakeholders. At other times it requires changing the balance of power in relationships through counselling, community mobilisation, advocacy and a range of other strategies all directed towards empowering persons with disabilities (Lorenzo 2008; Lorenzo & Cloete 2004; O’ Toole & McConkey 1995; WHO 2010).

An analysis of power holders, power relations and those who benefit from the current situation in families, communities and at broader levels, will alert the CDW to possible sources of resistance to change, whether these are political or traditional leaders, donors, families or persons with disabilities themselves. If persons with disabilities are included this equates to their capability to ‘do for themselves’, to contribute according to their capability to meet their own needs. It means a shift from charity to facilitating the optimal participation of persons with disabilities in their own development. Inclusion at a family level is essential to break down attitudinal and other barriers and to help families become the context in which persons with disabilities can develop their full potential (McKenzie & Müller 2006). Community disability workers facilitate the coordination of services across sectors to enable a supportive family life and community living.

## Conclusions and recommendations

The study confirms the relevance and value of community based rehabilitation, a strategy of community development that works towards the equalisation of opportunities, not only through rehabilitation, but also by addressing poverty and structural barriers, and also promoting the social inclusion of all persons with disabilities (WHO 2010). It confirms the importance of understanding and working with the concept of ‘disability’ from a social justice and development perspective, and not only the functional limitations related to an impairment. The interventions of CDWs need to document, target, measure and monitor the many barriers to inclusion and participation of persons with disabilities that are created, not by the impairments themselves, but by social barriers related to discriminatory attitudes, actions, relationships and power imbalances in the complex rural contexts in which they live.

The social inclusion of persons with disabilities requires changes in attitudes, actions and relationships in families and communities. For this reason, we advocate for the training and support of CDWs, to help them adopt a systems perspective of the community context and to develop an understanding of the power dynamics at play in and between systems – individual, family, organisation and community – and to learn how to influence them. Arguably this has been neglected in the training and support offered to CDWs. Moreover, the systems in which they work are often ill-equipped to respond to these challenges.

Donors should preferably be brought into the aforementioned partnerships with service providers and persons with disabilities, to deepen their understanding of the local context and to ensure that local organisations exercise more power in donor decision-making processes (Van Blerk 2005). Of potential value to CDWs and their employing bodies would be access to donor profiles and research into the attitudes and thinking that influence decisions about the allocation of both financial and non-financial resources.

The effective implementation of disability-inclusive development requires combined efforts, through partnerships and alliances of persons with disabilities, their families, organisations, donors, and relevant governmental and non-governmental services. However, an unanswered question that requires further research is, whether the promotion of such development in rural (and other) communities necessarily requires specialist community disability workers, or whether generic community development workers could be adequately trained and equipped for the challenge.

## Footnotes

1 Policy practice involves, inter alia, developing, analysing and refining policies that guide the operations of government and non-governmental organisations that have an impact on individuals, groups and communities (Homan 2004).
